# Fabrication and Mechanical Properties of Cr_2_AlC MAX Phase Coatings on TiBw/Ti6Al4V Composite Prepared by HiPIMS

**DOI:** 10.3390/ma14040826

**Published:** 2021-02-09

**Authors:** Muhammad Waqas Qureshi, Xinxin Ma, Guangze Tang, Bin Miao, Junbo Niu

**Affiliations:** 1 State Key Laboratory of Advanced Welding and Joining, Harbin Institute of Technology, Harbin 150001, China; waqasmse@hit.edu.cn; 2 School of Materials Science & Engineering, Harbin Institute of Technology, Harbin 150001, China; oaktang@hit.edu.cn (G.T.); 18b309004@stu.hit.edu.cn (B.M.); 19b309009@stu.hit.edu.cn (J.N.)

**Keywords:** MAX phase, HiPIMS, Cr_2_AlC, average target power, TiBw/Ti6Al4V composite

## Abstract

The high-power impulse magnetron sputtering (HiPIMS) technique is widely used owing to the high degree of ionization and the ability to synthesize high-quality coatings with a dense structure and smooth morphology. However, limited efforts have been made in the deposition of MAX phase coatings through HiPIMS compared with direct current magnetron sputtering (DCMS), and tailoring of the coatings’ properties by process parameters such as pulse width and frequency is lacking. In this study, the Cr_2_AlC MAX phase coatings are deposited through HiPIMS on network structured TiBw/Ti6Al4V composite. A comparative study was made to investigate the effect of average power by varying frequency (1.2–1.6 kHz) and pulse width (20–60 μs) on the deposition rate, microstructure, crystal orientation, and current waveforms of Cr_2_AlC MAX phase coatings. X-ray diffraction (XRD), scanning electron microscopy (SEM), and atomic force microscopy (AFM) were used to characterize the deposited coatings. The influence of pulse width was more profound than the frequency in increasing the average power of HiPIMS. The XRD results showed that ex situ annealing converted amorphous Cr-Al-C coatings into polycrystalline Cr_2_AlC MAX phase. It was noticed that the deposition rate, gas temperature, and roughness of Cr_2_AlC coatings depend on the average power, and the deposition rate increased from 16.5 to 56.3 nm/min. Moreover, the Cr_2_AlC MAX phase coatings produced by HiPIMS exhibits the improved hardness and modulus of 19.7 GPa and 286 GPa, with excellent fracture toughness and wear resistance because of dense and column-free morphology as the main characteristic.

## 1. Introduction

Among lightweight materials, titanium matrix composites (TMCs) are suitable candidates in automotive, aerospace, and military applications because of their excellent combination of mechanical properties and wear resistance [1]. The TiBw/Ti6Al4V composite is a type of discontinuously reinforced titanium matrix composite (DRTMC), in which reinforcement of TiB-whiskers (TiBw) was formed around the matrix of Ti6Al4V particles, forming a 3D quasi-continuous network architecture [2]. Because of this novel structure, TiBw/Ti6Al4V composite possesses superior isotropic properties to its counterpart, monolithic Ti-6Al-4V alloy [3]. The influence of process parameters on the properties of novel TiBw/Ti64 composite could be found in the literature [2,4,5,6,7]. TiBw/Ti64 composite shows superior mechanical properties at temperatures of 500–600 °C, but poor oxidation resistance due to the formation of unprotected TiO_2_ scale above the restricted temperature range [8,9]. Protective MAX phase films could play their part to enhance its oxidation and wear resistance further in the harsh environment.

M_n + 1_AX_n_ (known as MAX phases, where M is an early transition metal, A is an IIIA-or IVA group element, and X is either C or N, n = 1–3) phases are the nanolaminates ternary carbide and/or nitrides. These compounds have immense scientific and technological applications owing to their remarkable combination of metallic and ceramic attributes. The unique layered structure of MAX phases consists of a strong covalent M-X bond and relatively weak metallic M-A bond, which are responsible for their hybrid properties [10,11]. Typically, Cr_2_AlC is a type of the 211 MAX phases that exhibits excellent oxidation and hot corrosion resistance at elevated temperature owing to the formation of a continuous, well adherent, and inert Al_2_O_3_ protective layer [12,13]. Surprisingly, Cr_2_AlC MAX phase possesses the second-highest elastic modulus (358 GPa) after the Ta_2_AlC MAX phase and comparable thermal expansion coefficient (CTE $13.3\times 10^{-6}\;K^{-1}$) to many commercial alloys [14,15]. Therefore, Cr_2_AlC is considered a protective coating material for alloys’ servicing in high temperature and corrosive environments. Cr_2_AlC thin film was first synthesized by magnetron sputtering on the heated sample from three elemental targets in DC mode. The results showed that sputter power control for the individual target is complicated to get a strict stoichiometric composition range of chromium, aluminum, and carbon [16,17]. Recently, the two-step method accompanied by a compound target has been frequently adopted, in which deposition at room temperature followed by ex-situ annealing has proven to be more feasible [18,19,20]. However, a proper crystallization temperature is required to obtain the epitaxial or polycrystalline phase-pure MAX phase. The temperature lower than crystallization temperature results in competing phases such as inverse perovskite Ti_3_AlC [21], or a tripled layer structure (α-(Cr, Al)_2_O_3_/amorphous layer/Cr_2_AlC)) [22]. The minimal crystallization temperature of 450 °C [23], and later 370 °C [22], was reported for the formation of Cr_2_AlC film, which is far lower than that of Ti_2_AlC, Nb_2_AlC, and Ti_3_SiC_2_ coatings [24,25,26]. Moreover, the effect of sputtering power and bias voltage on microstructure and mechanical properties of Cr_2_AlC thin film was studied, and the results showed that the deposition rate and hardness are proportional to the sputtering power and bias voltage [27,28]. Phase-pure crystalline Cr_2_AlC films have been successfully deposited on M38G superalloy from a cost-efficient composite target through magnetron sputtering [19] and arc ion plating (AIP) [29], and it was claimed that film composition depends on elemental composition instead of the phase composition in the compound targets.

High power impulse magnetron sputtering (HiPIMS/HPPMS) is a new addition to the class of PVD techniques for thin film deposition, which utilizes ions or at least a large fraction of ionized species instead of neutrals during the deposition [30]. According to Anders et al. [31], HiPIMS is a pulse sputtering where the peak power density exceeds two orders of magnitude of the time-averaged power. In HiPIMS, a much higher power density is applied to the target during the short pulse on-time. Because of the high degree of ionization, HiPIMS has excellent potential to synthesize high order material such as the MAX phase and tailor the phase composition and structure, and hence the properties of thin films [32]. Fu et al. [33] studied the oxidation and corrosion behavior of Ti_2_AlC MAX films deposited through HiPIMS on the stainless-steel substrate. However, the application of HiPIMS in MAX phase thin film deposition has been limited so far [34,35,36]. The study on the influence of deposition parameters on film microstructure and properties is lacking. Thus, the HiPIMS technique needs to be further explored in the area of MAX phase thin-film synthesis, and a comparative study should be made with other conventional PVD techniques, i.e., dc magnetron sputtering (DCMS).

The motivation of this work is mainly focused on the potential of the HiPIMS technique to deposit Cr_2_AlC MAX phase coatings on network structured TiBw/Ti6Al4V composite in comparison with conventional DCMS. In this study, the influence of average power (P_ave_) at a different frequency and pulse width on film quality, phase composition, deposition rate, and roughness is comparatively addressed for each case in detail. The microstructural investigation of the as-deposited Cr-Al-C and annealed Cr_2_AlC films at different average power (P_ave_) was carried out by X-ray diffraction (XRD), scanning electron microscopy (SEM), and atomic force microscopy (AFM), and the mechanical properties were evaluated by the nanoindentation, Vickers hardness, and scratch test.

## 2. Experimental Details

### 2.1. Coating Deposition

**Figure 1:**
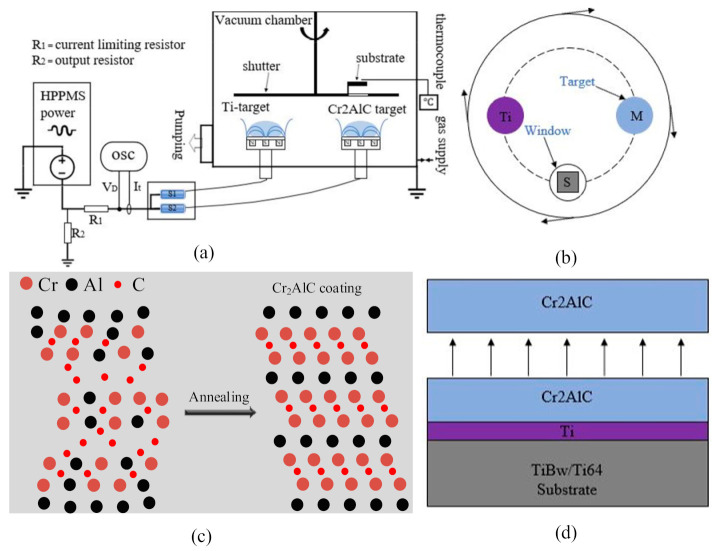
Schematic of the deposition process: (**a**) JGP560 sputtering system with high-power impulse magnetron sputtering (HiPIMS) power source; (**b**) arrangement of magnetron targets inside the vacuum chamber (top view), where “S” and “M” represent substrate and MAX compound target, respectively; (**c**) formation mechanism of Cr_2_AlC coatings followed by ex situ annealing; and (**d**) design of Cr_2_AlC coating system on TiBw/Ti64 substrate having a Ti buffer layer between Cr_2_AlC coating and substrate (arrows illustrate the thickness of deposited coating).

TiBw/Ti6Al4V composite with a novel network structure was used as a substrate material in this study. Samples in the dimension of ∅15×2 mm were cut from a large plate and were firstly ground with SiC paper, followed by mirror polishing with 0.5 μm diamond suspension. The deposition was done in a lab-scale JGP560 sputtering system (SKY Technology Development Co. Ltd., Shenyang, China) with base pressure lower than 6 $\times 10^{-4}$ Pa and CHANT (Shandong Zibo Changtai Electric Co. Ltd., Zibo, Zibo, China) HiPIMS power source was used for the deposition of Cr-Al-C coating. The target voltage (V) and the discharge current (I) were measured by a digital oscilloscope (RIGOL -DS1064B, China) with a voltage probe (Tektronix, model P-5100, Beaverton, OR, USA). Two unbalanced bipolar magnetron sources were placed inside the vacuum chamber with circular elemental Ti (99.99 at%) and compound Cr_2_AlC (2:1:1 molar ratio) targets with a dimension of ∅50×5 mm provided by Nanjing Materials Company. Before mounting to the substrate holder (distant at 60 mm from magnetron target), samples were thoroughly cleaned ultrasonically in an acetone and alcohol bath to remove the surface contaminations and dried in the air, respectively. The schematic diagram of the sputtering system and arrangement of targets along with the coating design is illustrated in Figure 1. For all coatings on TiBw/Ti6Al4V substrate, a Ti-layer is deposited as a diffusion barrier (buffer layer), as shown in Figure 1d. The sputtering deposition was carried out in a pure Ar (99.99%) atmosphere controlled by a mass flow controller, and the working pressure was maintained at 0.5 Pa, regulated by the main chamber valve. Prior to deposition, the substrates were plasma etched at −400 V DC voltage for 30 min to remove possible surface contaminants. Two groups of experiments were conducted by varying the frequencies and pulse widths at constant discharge voltage and duty cycle as, shown in Table 1, resulting in constant peak power (P_peak_), but different average power (P_ave_). The average power (P_ave_) and peak power (P_peak_) were calculated as follows:

(1) $$P_{\mathrm{ave}}=f.\int_{0}^{\tau }U(t).I(t)\mathrm{dt}$$

(2) $$P_{\mathrm{peak}}{=I}_{p}\times U_{s}$$

where U(t) and I(t) are the discharge voltage and current in terms of time; and f, τ, I_p_, and U_s_ are frequency, pulse width, peak current, and stable target voltage, respectively.

During the deposition, a shutter with a window parallel to targets was installed between the magnetron target and substrate, which allow the sputtering from the individual target and protect other targets from surface contamination. An around 1.5 μm thick Ti-layer was introduced as a diffusion barrier (parameters not shown here for simplicity) for better adhesion between Cr-Al-C coating and substrate and to limit the interdiffusion of Al into the substrate during annealing. The sample was first rotated to the elemental Ti-target for deposition, then moved to the Cr_2_AlC compound target. Moreover, no external heating and bias were applied throughout the deposition process, and the gas temperature around the substrate was monitored through the thermocouple placed at 10 mm to the substrate. For comparison, Cr_2_AlC coating was deposited through DCMS on Si (111) wafer without a Ti buffer layer, because Ti is difficult to be sputtered owing to the low deposition rate at the sputtering power (P_ave_ = 112 W) used for Cr_2_AlC coatings. A two-step approach was adopted: deposition of Cr-Al-C coatings on bare composite at room temperature followed by subsequent ex situ annealing to transform amorphous Cr-Al-C into crystalline Cr_2_AlC MAX phase. As-deposited Cr-Al-C coatings were ex situ annealed in a high vacuum annealing furnace (VTHK-550/Beijing Technol Science Co.Ltd., Beijing, China) at 650 °C holding for 1 h isothermally, and substrates were then furnace cooled after annealing. Figure 1c demonstrates the annealing effect on as-deposited coatings, indicating that the amorphous coatings crystallize into Cr_2_AlC MAX phase coatings after annealing.

### 2.2. Characterization Methods

The crystal structures of as-deposited and annealed coatings were characterized using an X-ray diffractometer (Panalytical Analytics Instruments-Netherlands) using the grazing incidence X-ray diffraction (GIXRD) method with Cu-Kα radiation (λ = 1.54 $A^{0}$). The data were collected between the 2θ angle range from 10° to 90° at a grazing angle of 0.5° with a step size of 0.04°. The chemical composition and microstructure of coatings were examined by scanning electron microscopy (SEM, Zeiss DSM-960A, Jena, Germany) equipped with an energy dispersive X-ray spectrometer (EDX). The deposition rate was calculated from the measured coatings thickness observed in SEM micrographs. The deposition rate corresponds to the ratio between the coating’s thickness to deposition time with the unit of nm/min. The surface roughness was measured by the atomic force microscopy (AFM, Bruker, Karlsruhe, Germany) in a tapping mode with a scan area of 1 μm×1 μm. The surface hardness and Young’s modulus of the coatings were measured by Berkovich nano-indenter (UMIS-2000, Red Bluff, CA, USA) by adjusting the indentation depth at 1/10th of the total thickness of the coating to avoid the substrate effect. For each coating, six replicate indentations were performed to evaluate the average hardness (H) and Young’s modulus (E). Furthermore, adhesion between coating and substrate was evaluated by performing a scratch test using a scratch tester (MFT-4000, Lanzhou, China). A scratch test length of 8 mm was used at a loading rate of 10 N/min up to 20 N. The scratched morphology and Vickers indents were analyzed using SEM. Vickers hardness tests were performed on a Vickers automated hardness tester (TUKON-2500, Cracow, Poland) with a normal load of 300 g, having a dwell time of 10 s to test the fracture toughness of coating. The fracture toughness of the coatings was calculated using the Anstis relation [37]:

(3) $$K_{\mathrm{IC}}=0.016\times {\left(\frac{E}{H}\right)}^{1/2}\left(\frac{P}{C^{3/2}}\right)$$

where K_IC_ is the fracture toughness, H is the hardness, E is the elastic modulus, P is the hardness testing load, and c is the average crack length from the center of indent to the crack tips measured by SEM.

## 3. Results and Discussion

### 3.1. HiPIMS Discharge Characteristics

**Figure 2:**
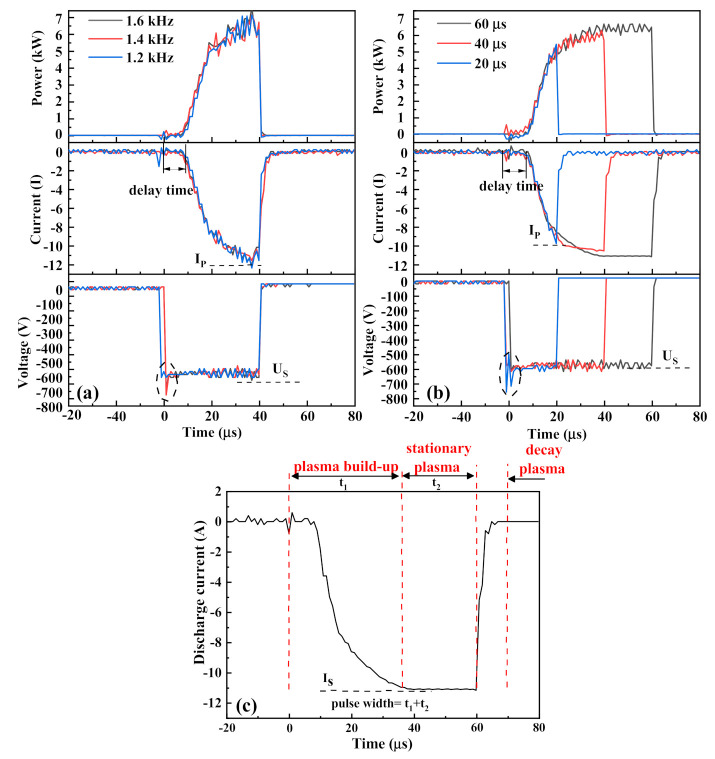
Voltage and discharge current (I–V) oscillogram in HiPIMS at (**a**) different frequencies (1.2–1.6 kHz); (**b**) different pulse widths (20–60 μs); and (**c**) three discharge current operation regimes at 60 μs, where pulse width (τ) = plasma build-up time (t_1_) + stationary plasma time (t_2_).

Cr_2_AlC coatings were prepared at different combinations of frequency and pulse width at constant discharge voltage and duty cycle, which results in variable average power (P_ave_) depending on plasma characteristics. The detailed parameters for deposition are shown in Table 1. The applied quasi-constant target voltage and discharge current evolution (I–V curve) in unipolar HiPIMS mode is depicted in Figure 2. It is observed that target voltages are in a rectangular shape, which is correlated with a linear increase in discharge current in a triangular shape regardless of pulse width and frequency. It is worth noting that the discharge current waveform at different parameters in HiPIMS is of utmost importance to determine plasma characteristics and deposited coatings’ properties. Figure 2a shows the one pulse discharge current and a voltage waveform (I–V curve) of the ∅50 mm Cr_2_AlC target at various average powers. The average power was altered by changing the frequency from 1.2 kHz to 1.6 kHz at a constant voltage of 600 V and a pulse width of 40 μs. It is seen that there is an oscillation in discharger voltage caused by plasma instabilities at pulse initiation, which is ascribed as the ignition phase (the black circle in Figure 2). Later on, it is almost in steady-state throughout the pulse denoted as U_s_. On the other hand, the discharge current increases linearly to a peak current of ~−12 A (referred to I_p_), followed by the triangular current waveform. It is noticeable that the characteristic delay time during which discharge current lagging behind the voltage pulse by 10 μs can be ascribed as the ignition time (delay time) of glow discharge in magnetron sputtering (MS). Voltage and the discharge current oscillogram of Cr_2_AlC target at different pulse width from 20 μs to 60 μs are shown in Figure 2b. The discharge current increased linearly to its peak level of ~−10 A and ~−11 A at 40 μs and 60 μs pulse width, respectively, and then tends to be stable in the remaining part of the pulse width.

The peak power (P_peak_) and ionization of the target material are correlated with the current amplitude in discharge. The peak current (I_p_) increases with the increase in pulse width, resulting in higher peak power and plasma ignition time (delay time). According to Musil et al. [38,39], glow discharge current in the triangular waveform can be divided into three operational regimes: (i) plasma build-up regime, (ii) stationary plasma regime, and (iii) decaying plasma regime. On the other hand, Gudmundsson et al. divided the current waveform into four parts [40]. The stationary plasma regime is directly analogous to the duration of discharge current in the plasma stabilization regime. It is observed that the stationary plasma regime for the wider pulse is larger. Therefore, the plasma stabilization regime is absent for the short pulse width (20 µs), while the stationary plasma regime appears when the pulse width is higher than 20 µs. The discharge current at 20 µs pulse width increases linearly to a maximum value following the plasma build-up portion and drops abruptly to zero, making the decaying plasma section without stationary plasma regime, Figure 2b. In contrast, the pulse width larger than 20 µs allows the saturation of discharge current and establishment of a steady-state plasma [39]. The discharge current becomes stable at ~−10 A and ~−11 A at a pulse width of 40 µs and 60 µs, respectively. The three operational regimes within the current waveform during the glow discharge magnetron sputtering at 60 μs are shown in Figure 2c. The role of the stationary plasma regime is crucial for the average power (P_ave_) because the discharge current is maximum and stable in this regime. So, according to the equation P(t) = U(t) × I(t), the average power is always higher for a wider pulse width at a constant voltage and frequency (see Table 1). It is worth noticing that the increase in pulse width or frequency while keeping the remaining parameters constant increases average power. So, from now, an increase in pulse width or frequency should be considered as an increase in average power for better understanding.

### 3.2. Microstructure and Phase Composition

**Figure 3:**
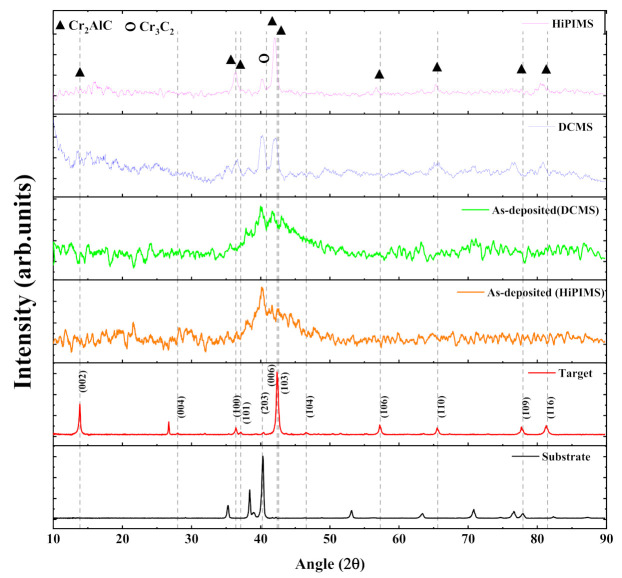
Grazing incidence X-ray diffraction (GIXRD) patterns for the coatings as-deposited and annealed coatings by dc magnetron sputtering (DCMS) (P_ave_ = 112 W) and HiPIMS (P_ave_ = 110 W).

Grazing incidence XRD patterns for as-deposited and annealed Cr_2_AlC coatings deposited by HiPIMS and DCMS on TiBw/Ti64 composite are presented in Figure 3. For comparison, the bulk Cr_2_AlC target and substrate spectra are also presented. The target material is mainly composed of the Cr_2_AlC MAX phase with very weak chromium carbide peaks (Cr_3_C_2_) as an impurity. There is an amorphous hump at $2\theta \approx 42^{{}^{\circ}}$ for as-deposited Cr-Al-C coatings along with other weak peaks, indicating that the coatings have an amorphous structure and have not been crystallized yet. It also suggests that high atom activity is required to form a high ordered MAX phase crystal structure. According to previous findings, crystallization of amorphous Cr-Al-C coatings deposited from the compound target and elemental target could occur at 500 °C and 650 °C, respectively [22,41]. Thus, in the present work, annealing was carried out at 650 °C for 1 h to obtain crystalline coatings, and XRD patterns changed significantly compared with those of as-deposited coatings. The coatings deposited by the HiPIMS and DCMS exhibit sharp peaks, indicating the transformation from amorphous to crystalline structure taking place, and XRD peaks are in correspondence with the Cr_2_AlC MAX phase (PDF # 29–0017). The unknown peak at 26° in the Cr_2_AlC bulk target is absent in the coatings’ patterns obtained after annealing. The presence of chromium carbide in XRD results indicates that the coatings’ composition somewhat differs from the target, resulting from preferred aluminum resputtering during the deposition process [34]. Moreover, the Cr-Al-C phase diagram confirms the coexistence of intermediate phases (AlC_2_, Cr_7_C_3_, Cr_3_C_2_) with Cr_2_AlC MAX phase if the stoichiometric ratio of Cr/Al/C deviates from 2:1:1 [20,42]. Therefore, XRD patterns obtained from coatings after annealing contain a relatively sharp peak of Cr_3_C_2_ (203) (PDF # 71−2287) at 2θ≈40.20°. Moreover, the Cr_2_AlC (106) peak is absent in the coating deposited by DCMS, and the Cr_3_C_2_ (203) peak in DCMS is much stronger than that of HiPIMS.

**Figure 4:**
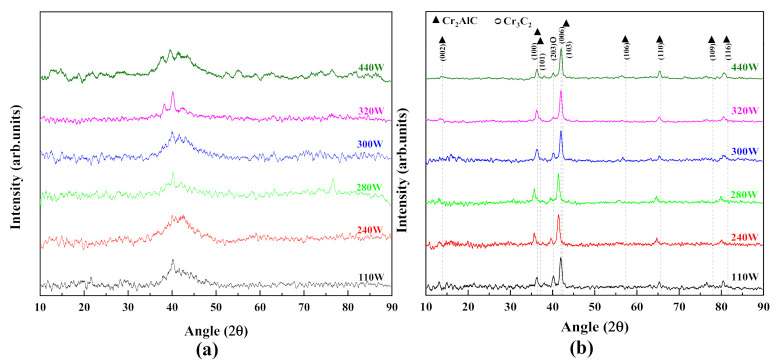
Grazing incidence XRD patterns of (**a**) as-deposited and (**b**) annealed Cr_2_AlC MAX phase coatings synthesized at different average power.

Similarly, GIXRD patterns of as-deposited and annealed coatings prepared by HiPIMS at different average power are shown in Figure 4. The as-deposited Cr-Al-C coatings possessed an amorphous structure along with a significant amount of Cr_2_C_3_, according to 2θ around 42°, similar to that of coatings prepared by DCMS (see Figure 4a). It is observed from Figure 4b that the as-deposited coatings showed some degree of crystallization because the XRD spectra of as-deposited coatings show some peaks along with an amorphous hump. However, the temperature during the deposition could not reach the crystallization temperature of Cr_2_AlC MAX phase coatings. This result is consistent because the coating prepared by magnetron sputtering without intentional heating of the substrate was amorphous [43]. It is known that the MAX phases crystallize in the hexagonal crystal structure (space group P6_3_/mmc), and [0001] basal plans are the most stable orientation because of the lowest surface energy [44]. Experimentally, the epitaxial or single crystalline MAX-phase film can also be synthesized, in which crystals only grow along the [0001] basal plane [45,46,47]. However, the coatings after annealing show various diffraction peaks other than the [0001] basal plane. It can be seen that the coatings deposited by DCMS are almost the same as those by HiPIMS; however, after annealing, the peak (006) becomes narrower and sharper, which indicates that films deposited by HiPIMS can easily form the preferred orientation, as reported previously [20,43]. The (006) and (101) reflexes exclusively belong to the ordered MAX phase, and a change in these reflections shows how disordered solid solutions transformed into ordered MAX phase after annealing. It is observed that the Cr_2_C_3_ peak decreases with the increase in average power after annealing, indicating that high HiPIMS power favors the Cr_2_AlC MAX phase. It may be because of the depletion of deposited Cr and Al, because of high ionization in HiPIMS, and will affect the Cr/Al ratio (discussed in Figure 7a).

It is noted that MAX phase peaks after annealing shift towards a lower Bragg’s diffraction angle as compared with that of the target material, which can have many origins, such as atomic ordering, change in chemical composition, change in crystallize size, lattice strain, and stresses arising due to mismatching at the interface of coating and substrate or due to thermal annealing. Therefore, lattice parameters, dislocation density, and lattice strain are calculated by calculating the d_hkl_ from Bragg’s law (2dsinθ=nλ) using the XRD data, and the obtained data are tabulated in Table 2. For comparison, lattice parameters for the Cr_2_AlC MAX phase are calculated by first-principles calculation using generalized gradient approximation (GGA-PBE) according to our previous work [48]. It is observed that the calculated lattice parameters for Cr_2_AlC coatings are in good agreement with the theoretical and experimental results for the Cr_2_AlC bulk and coatings. Moreover, the grain size of the coatings obtained at different average power ranges from 12 to 15 nm, which is smaller than that of bulk material, as expected. It is also observed that there is some degree of lattice strain in the coatings deposited by HiPIMS and DCMS, which is the possible reason for the peak shift towards the lower angle in the XRD patterns.

**Figure 5:**
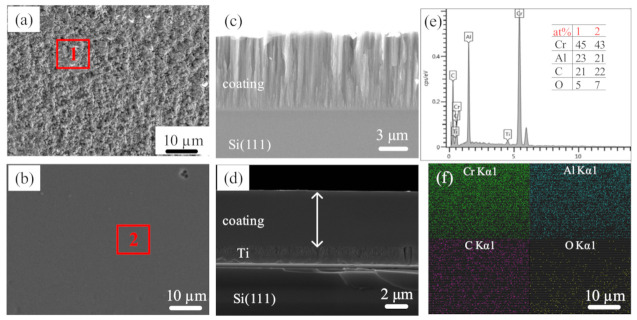
Surface and cross-sectional morphology of the Cr_2_AlC coatings deposited by (**a**), (**c**) conventional DCMS (*p* = 112 W) without a Ti buffer layer and (**b**), (**d**) HiPIMS (P_ave_ = 110 W) with a Ti buffer layer; (**e**) EDS spectrum and quantification results of red points in (**a**,**b**); and (**f**) EDS elemental mapping of (**b**). Quantification error within 5%.

The surface and cross-sectional morphology of the Cr_2_AlC MAX phase coatings deposited by conventional DCMS and HiPIMS is shown in Figure 5. For better cross-sectional visibility, coatings were deposited on Si (111) substrate without external heating and bias voltage under the Ar-environment at 0.5 Pa. Figure 5a,c represents the surface and cross-sectional morphology of Cr_2_AlC MAX coatings without a Ti buffer layer deposited by DCMS. It is observed that the coating surface has a granular structure, which is a typical structural morphology of Cr_2_AlC coatings prepared by DCMS [22,27]. The cross-sectional morphology of coatings consists of columnar grains with a column thickness of ≈250–500 nm. The column grains’ growth angle in the Cr_2_AlC coatings is affected by the bias voltage applied to the substrate during the DCMS. It is found that, with the increase in bias voltage, the column growth angle decreases from the right angle [27]. In this work, the growth of the column is perpendicular to the substrate as no bias voltage is applied to the substrate, which agrees with the results mentioned earlier. The equiaxed or column-free morphologies of Cr_2_AlC MAX phase coatings are required because the columnar grain of Cr_2_AlC coatings has poor oxidation resistance due to inward diffusion of oxygen through the columnar grain boundaries, which cause the internal oxidation of the Cr_2_AlC coatings [27]. A recent study showed that the oxidation resistance of Cr_2_AlC coatings could be enhanced by changing the coating’s morphology from columnar to equiaxed [51]. Furthermore, the hardness, wear-resistance, and fracture toughness of V_2_AlC MAX phase coatings have been improved by increasing the columnar grain boundaries [52]. Thus, the oxidation resistance and mechanical properties of MAX phase coatings can be tailored by changing the grain size and coating morphology.

One of the main characteristics of HiPIMS includes high ionization of target material that can be achieved at target peak power. The high ionization of target material can modify the deposited layer microstructure, and hence their properties. It is reported that modulating the ion-to-atom ratio (by changing pulse width and/or frequency) can change the microstructure of CrN and TiN films from columnar to fully dense along with improved hardness and roughness with excellent adhesion [53,54]. Figure 5b,d represents the surface and cross-sectional morphologies of Cr_2_AlC MAX phase coatings with a Ti buffer layer deposited by HiPIMS. The surface and cross-sectional morphology of coatings is significantly different from that of DCMS. It is observed that the coating surface possesses a smooth and crack-free morphology with finer grain size compared with that deposited by DCMS. Similarly, the cross-section microstructure of Cr_2_AlC MAX phase coatings deposited by HiPIMS possesses a smooth and glassy morphology with a fully dense structure without columns. The coatings prepared by both DCMS and HiPIMS without or with a Ti buffer layer are crack-free and well adherent to the substrate material, and interfaces between the Cr_2_AlC/Si(111) by DCMS and Cr_2_AlC/Ti/Si(111) by HiPIMS are clear and distinct. Our results are consistent with the Cr_2_AlC coatings deposited by DCMS without a buffer layer [27].

In this study, the column-free and denser microstructure of Cr_2_AlC MAX phase coatings deposited by HiPIMS is exclusively different from the structures reported previously [28,32,55,56,57,58]. The improved microstructure of the Cr_2_AlC MAX phase coatings is responsible for enhanced hardness and modulus (discussed later). According to the structural zone model (SZM) by Thornton, the deposited Cr_2_AlC coating structure belongs to zone-T [59]. The energy dispersion analysis of the coatings shows that the ratio of Cr/Al in at. % for DCMS and HiPIMS is ${\left(Cr/Al\right)}_{DCMS}=1.91$ and ${\left(Cr/Al\right)}_{HiPIMS}=2.01$, which is acceptable as the value of Cr/Al for the stoichiometric Cr_2_AlC is 2. It is also observed that the deposited Cr in HiPIMS is depleted as compared with deposited Cr in DCMS in coatings owing to the high degree of ionization. Moreover, the elemental mapping was enriched in Cr, Al, and C elements and is equally distributed throughout the coatings’ surface with a small amount of O element, which could be from the impurity of the target or due to exposure of samples to ambient for ex situ annealing (see Figure 5f).

**Figure 6:**
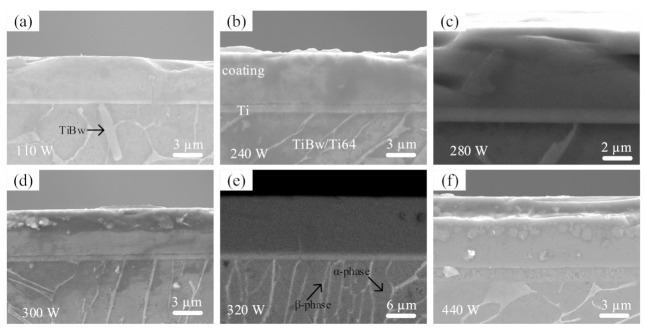
SEM micrographs of the Cr_2_AlC MAX phase coatings deposited by HiPIMS on TiBw/Ti6Al4V composite at P_ave_ (**a**) 110 W, (**b**) 240 W, (**c**) 280 W, (**d**) 300 W, (**e**) 320 W, and (**f**) 440 W.

Figure 6 presents the cross-sectional morphology of the coatings deposited by HiPIMS on the TiBw/Ti6Al4V at different average power. It is observed that all the coatings are dense, free from cracks and voids, and tightly bonded to the substrate without columnar growth. The column-free morphology of coatings observed here in this study is distinct from the Cr_2_AlC coatings reported previously [12,32,58]. Interestingly, the coatings possess duplex structures regardless of deposited parameters. The Ti-layer (light grey) is much thinner than the outer Cr_2_AlC layer (dark grey), as expected. The duplex morphology consists of outer Cr_2_AlC-layer/Ti-layer and Ti-layer/substrate interfaces that are clear, distinct, and well bonded to each other. Featured grains are non-distinguishable at this scale and are believed to be polycrystalline Cr_2_AlC MAX phase, as shown in GIXRD spectra in Figure 4. The thickness of the Cr_2_AlC coating was according to the deposition parameters, and the deposition rate was measured from SEM micrographs by taking an average of ten measurements. The purpose of the diffusion barrier (buffer layer) between the coating and substrate proved to be helpful to prone elemental interdiffusion (especially “A” elements in MAX) from the coating to substrate [60]. A double layer of diffusion barrier has been proposed to stop the decomposition of Cr_2_AlC and Al diffusion into the Zr substrate up to 1000 °C [61]. The Cr_2_AlC/Ti/substrate interfaces are distinct and clear for all coatings, which indicates that there is no interdiffusion of Al occurring between the Cr_2_AlC coating and substrate along the Ti diffusion barrier during annealing. Furthermore, all the coatings deposited at different average power in HiPIMS show a similar morphology. The microstructure of TiBw/Ti6Al4V substrate consists of the darkish and white phase corresponding to equiaxed α and intergranular β phase, and needle-like TiB-whiskers (pointed by black arrow) are also shown.

**Figure 7:**
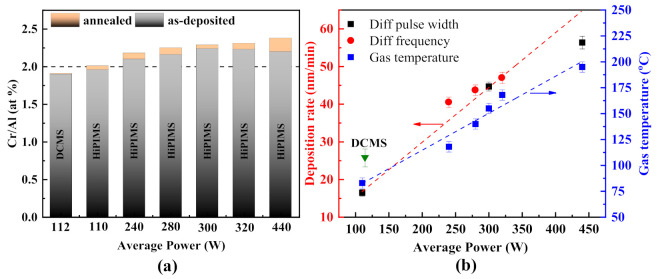
(**a**) Chromium to aluminum atomic ratio (Cr/Al) of the coatings deposited by DCMS and HiPIMS and (**b**) deposition rate and gas temperature around the substrate at different average power in HiPIMS (▼is the deposition rate for Cr_2_AlC coating by DCMS for comparison).

The Cr/Al atomic ratio of the coatings is presented in Figure 7a. The horizontal line shows the stoichiometric value for the Cr_2_AlC Max phase. The Cr/Al ratio for as-deposited and annealed coatings grown by DCMS $\left(P=112\;W\right)$ and HiPIMS $P_{ave}=110\;W$ is within 5% of the stoichiometric ratio. This ratio increases with the increase in average power in HiPIMS. The observed Cr/Al ratio in the annealed specimen is slightly higher than that of as-deposited coatings in each case, indicating that the deposited Cr and Al are depleted owing to high ionization in HiPIMS or preferred aluminum resputtering during the deposition process [34]. Moreover, Al loss is more significant in the annealed coating, which indicates that the Al is diffused out from the coating surface during the ex situ annealing because the 650 °C annealing temperature nearly equals the melting point of Al or vaporized into the furnace environment during the high vacuum annealing process.

The deposition rate and gas temperature at different average power are shown in Figure 7b. For comparison, the deposition rate of Cr_2_AlC coatings deposited by DCMS is also presented. Although the deposition rate of DCMS is almost double that of HiPIMS at the same level of power input, there is a limitation in the increase in deposition power, which leads to overheating and, consequently, damage of sputter magnetron. This drawback is overcome by HiPIMS, in which much higher power densities are applied to the target during the short pulse on-time, and overheating or damage to sputter magnetron is avoided. The average power increased by increasing pulse width and/or frequency; consequently, the deposition rate increases linearly. With the increase in pulse width and/or frequency, high ionization of target material occurred due to substantial ion bombardment on the target surface. This leads to more ejection of target atoms from the target surface, resulting in a higher deposition rate. According to the equation $P\left(t\right)=U\left(t\right)\times I\left(t\right)$, the average power increases significantly by increasing the pulse width rather than the frequency. Therefore, the deposition rate improved more effectively at a wider pulse width (black points) compared with the higher frequency (red points). The highest deposition rate achieved is 56.3 nm/min at an average power of 440 W. However, the deposition rate at $P_{ave}$ = 440 is below the linear fit line, indicating that the deposition rate was comparably low at 60 µs pulse width. The decrease in the deposition rate at 60 µs could be because of the yield effect and peak amplitude of discharge current caused by self-sputtering and working gas recycling (referring to the recycling of the ions of the inert working gas) [62]. Moreover, other factors like the ionic species effect and transport effect significantly influence the deposition rate in HiPIMS when the pulse width is higher than 50 μs [63]. However, it was found that the impact of self-sputtering and gas rarefaction can be omitted by applying a shorter pulse width or using bipolar pulse mode. It is interesting to see that the gas temperature around the substrate is affected by the average power. The gas temperature monitored at the end of the deposition process goes up from 83 °C to 195 °C with an increase in average power due to the higher bombardment of ionized particles per unit time.

**Figure 8:**
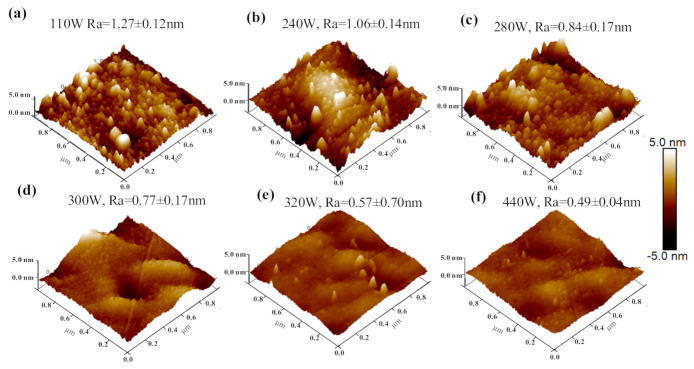
Atomic force microscopy (AFM) images of the Cr_2_AlC coatings deposited by HiPIMS at different average power: (**a**) 110 W, (**b**) 240 W, (**c**) 280 W, (**d**) 300 W, (**e**) 320 W, and (**f**) 440 W.

Further, the surface morphology of the surface of the Cr_2_AlC coating was examined by AFM. The coating roughness significantly affects the tribological performance and corrosion resistance of the coatings under service [64,65]. From the tribological point of view, friction depends on the deposited coating’s surface roughness, and a smoother surface gives a lower friction coefficient (COF) [66]. In HiPIMS, surface properties of the deposited coatings are tailored by changing the average power. The 3D surface morphology of Cr_2_AlC coatings deposited on Si wafer by HiPIMS at different average power is presented in Figure 8. It is worth mentioning that surface topography was conducted on the as-deposited samples because post-annealing samples possessed almost a similar morphology, which could be seen from Figure 6. The surface roughness of deposited coatings decreases with an increase in average power in HiPIMS. The minimum average roughness (R_a_) observed is 0.49 nm at an average power of 440 W. The energy of the ion of the sputtered material plays an essential role in the surface roughness and morphology of film [67]. When average power is higher, ionization and temperature during the deposition are elevated, favorable for sputtered atoms to diffuse on the substrate with reduced roughness. Similarly, at low average power, ionization is low, thus loose Cr_2_AlC coatings with relatively high roughness are observed.

### 3.3. HiPIMS Surface Defects

**Figure 9:**
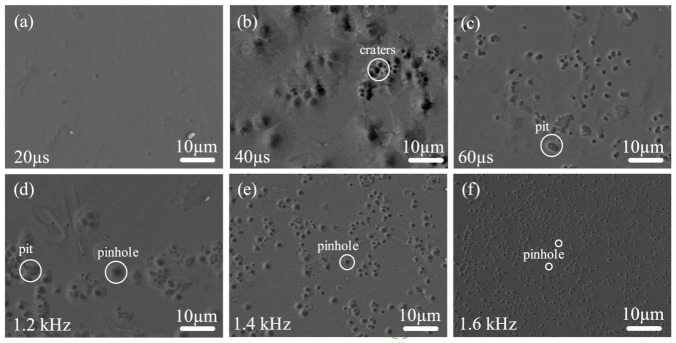
Surface morphology of defects on Cr_2_AlC coating surface produced during HiPIMS deposition by changing (**a**–**c**) pulse width, and (**d**–**f**) frequency.

HiPIMS is a relatively new addition to the class of PVD techniques for thin-film technology compared with DCMS. The major difference between HiPIMS and conventional DCMS is the operation mode and more control over deposition parameters. There is a high degree of plasma density and a high degree of ionization of sputtered material in HiPIMS, while in DCMS, sputtered material mainly consists of neutral species. Therefore, HiPIMS can produce a better quality of coatings with improved hardness, roughness, and adhesion [68,69]. It has been found that no deposition process can produce coatings that are entirely free from defects. The surface morphology of the Cr_2_AlC coatings deposited on TiBw/Ti6Al4V composite by HiPIMS is shown in Figure 9. It is observed that the coating surface smooth, dense, and free from cracks, but there are defects on some areas of the surface. These defects are divided into two categories. Firstly, the defects produced during increasing the average power by increasing the pulse width are presented in Figure 9a–c. Secondly, the defects produced during the increase in average power by increasing the frequency (see Figure 9d–f). Figure 9a represents the smooth surface and is free from any defect in the coating deposited at a pulse width of 20 µs $\left(P_{ave}=110\;W\right)$. When the pulse width is increased, craters and pits having a diameter of microns are observed on the coating surface (Figure 9b,c). These craters’ diameters were much smaller than those mentioned in some of the literature [70]. The size and area distribution of these defects are tabulated in Table 3. Similarly, when the average power in HiPIMS increases by increasing the frequency (1.2–1.6 kHz), the pinhole type of defect observed of the surface and pinhole defect diameter decreases with an increase in frequency.

The formation of these craters might be due to the occasionally arcing when a sudden increase in current and decrease in discharge voltage occurred during the deposition process. Because of this, microscopic debris target material was introduced on the surface of the substrate, which may produce an explosive eruption on the films during sputtering deposition. Although the unipolar HiPIMS power supply used in this study is equipped with arc detection and its suppression (deposition process halt when an arc is spotted) technology, but there has always been a delay in suppression after detection of an arc, which leads to plasma instability. The discharge plasma stability could be achieved by utilizing bipolar-pulse in which a small positive potential pulse was applied to the target during the pulse-off time to prone the arcing [71].

### 3.4. Mechanical Properties

**Figure 10:**
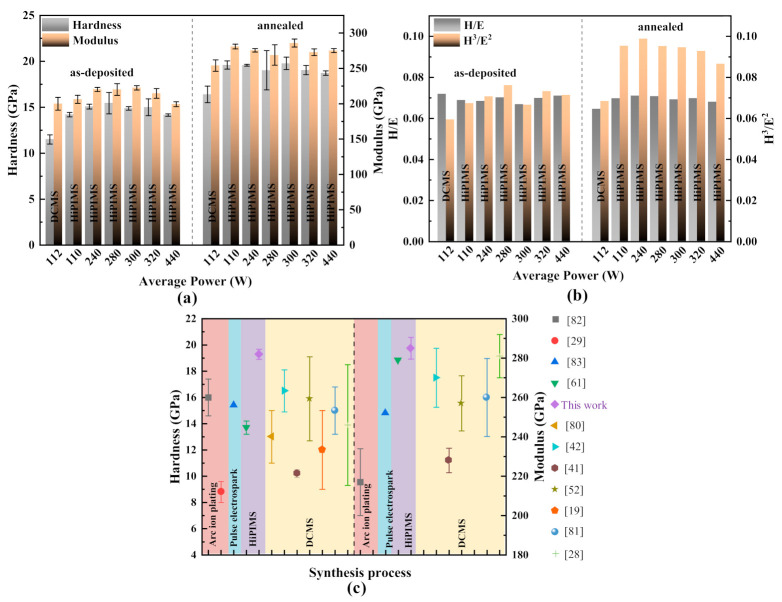
(**a**) The hardness (H) and elastic modulus (E), (**b**) H/E and H^3^/E^2^ value of Cr_2_AlC MAX phase coatings before and after annealing, and (**c**) comparative results of hardness and elastic modulus of Cr_2_AlC MAX phase coatings of our work and the references [19,20,27,28,29,41,58,77,78,79,80].

The hardness (H) and elastic modulus (E) of Cr_2_AlC MAX phase coatings before and after annealing deposited by DCMS and HiPIMS are illustrated in Figure 10a. The hardness and modulus of coatings are useful to predict sustainability wear protection of coatings, while modulus (E) represents the distribution of a given load on a larger area. In this study, the influence of average power on hardness and modulus of Cr_2_AlC MAX coatings deposited by HiPIMS compared with that of DCMS is the focus. It is noted that all the as-deposited coatings prepared either by DCMS or HiPIMS show relatively low indentation hardness (11.5–15.5 GPa) deposited at different average power. The low hardness is because of the amorphous nature of coatings before annealing. There is significant improvement in hardness and modulus observed after ex situ annealing due to crystallization of the Cr_2_AlC MAX phase. At different deposition power, the crystallized Cr_2_AlC MAX coatings show almost identical hardness ranging from 18 GPa to 20 GPa and elastic moduli between 270 GPa and 285 GPa, respectively. However, the obtained hardness of coatings is nearly double that of the TiBw/Ti6Al4V composite (5–8 GPa) used as a substrate [72]. Figure 10b illustrates the H/E and H^3^/E^2^ ratio of the Cr_2_AlC coatings in function with the deposition power in HiPIMS. The H/E ratio represents the resistance of coating against elastic strain to failure, and the H^3^/E^2^ ratio shows the resistance to plastic deformation [73,74]. It is observed that the H/E ratio changes slightly from 0.066 to 0.071, while the H^3^/E^2^ ratio is increased monotonously after annealing the coatings. All the coatings showed a nearly identical H/E and H^3^/E^2^ ratio, and the maximum value of the H^3^/E^2^ ratio is 0.098 for the coatings deposited at a power of 240 W. It is known that the high value of H/E can delay elastic to failure and a high H^3^/E^2^ indicates an excellent resistance to crack deformation and its propagation. The coatings produced by HiPIMS showed better wear resistance compared with DCMS. The column-free and dense microstructure of Cr_2_AlC MAX phase coatings benefits the excellent hardness and modulus along with high resistance to plastic deformation as compared with coatings prepared by conventional DCMS.

Figure 10c represents the comparative results of hardness and modulus of Cr_2_AlC coatings deposited in this study and other references. It is observed that the hardness and modulus of coatings prepared by HiPIMS show the highest values as compared with the Cr_2_AlC coatings prepared through DCMS and other PVD techniques (see references in Figure 10c caption). The hardness and modulus values of 19.29 GPa and 285 GPa for Cr_2_AlC coatings were observed in this study, which are comparatively better than the highest values observed in the literature so far. During the deposition in HiPIMS, high plasma density and high ionization of target material are produced, and high quality of coatings with improved microstructure is produced. The plasma characteristics are different in HiPIMS and DCMS, which have a significant influence on coating microstructure and Cr_2_AlC phase formation; hence, they can affect the mechanical properties. Moreover, the coating with an average grain size of tens of nanometers and large grain boundaries at larger density could also be the reason for increased hardness compared with bulk samples (where the grain size is in micrometers or larger and fewer grain boundaries), according to the Hall–Patch equation. This fact could be understood from the findings of Kooi et al. [75] and Emmerlich et al. [76], which proposed that the probability of kink formation during the indentation load application is responsible for increased hardness. Deposition by HiPIMS produced smooth, dense, and column-free Cr_2_AlC coatings as compared with coatings deposited by DCMS. Furthermore, the obtained elastic modulus value is in good agreement with the value of the reported Cr_2_AlC thin-film [77].

**Figure 11:**
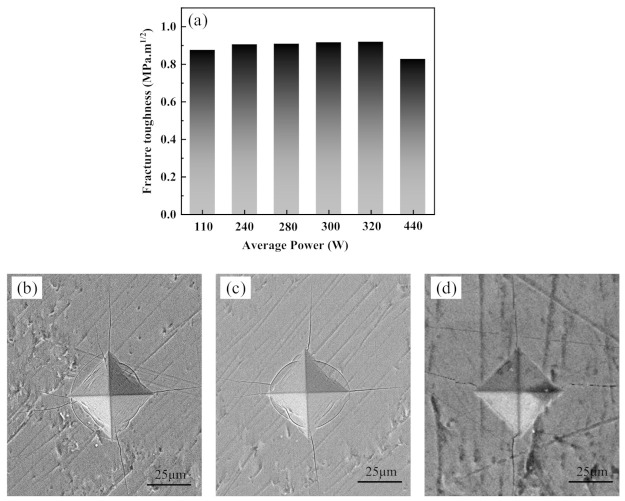
(**a**) Fracture toughness of the Cr_2_AlC MAX coatings deposited at different average power in HiPIMS and SEM micrographs of indentation formed for the coatings at P_ave_ of (**b**) 110 W (**c**) 300 W, and (**d**) 440 W.

In order to gain insight into the fracture toughness of the Cr_2_AlC coatings, the Vickers indentation was performed. Fracture toughness is one of the essential mechanical properties of the material, representing the ability to resist crack propagation during deformation up to fracture [73]. Figure 11a shows the fracture property of the Cr_2_AlC MAX phase coating determined by the Vickers indentation technique. Figure 11b–d illustrates the formed indentation shape and cracks expanded along the radial and axial direction for the coatings deposited at an average power of 110, 300, and 440 W, respectively. It is observed that fracture toughness of coatings showed almost identical results (≈0.9 MPa·m^1/2^) at different average power of the deposition process. These values correspond with the previous H/E ratio of coatings because all of the coatings showed similar morphology.

**Figure 12:**
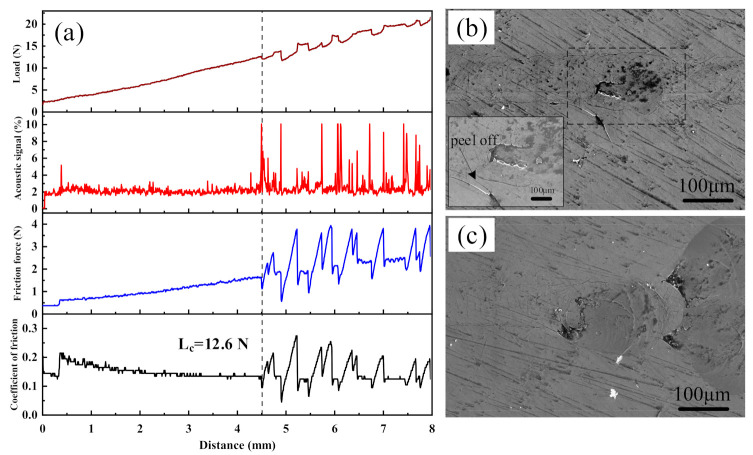
(**a**) Friction coefficient (COF) vs. lateral displacement curve combined with friction force and acoustic emission of Cr_2_AlC coating during the scratch test, SEM micrograph of (**b**) crack formation at critical load L_c_ (inserted micrograph shows the coating peel off), and (**c**) scratch at maximum load.

To further understand the integrity of the mechanical properties of Cr_2_AlC MAX phase coating on the TiBw/Ti6Al4V composite deposited by HiPIMS, the scratch test was performed, and a critical load (L_c_) of 12.6 N was observed, which is relatively better than that of coatings prepared by DCMS [81]. The Cr_2_AlC coatings showed a relatively low friction coefficient before forming a through-thickness crack (See Figure 12a). The coatings’ morphology along the scratch line was observed through SEM to evaluate the mechanical integrity of the Cr_2_AlC coating. Figure 12b shows the SEM micrograph at the critical load. It is observed the coating is peeled off, and the formation of a through-thickness crack was observed. Before the formation of a through-thickness crack, the coating exhibits the conformal cracking mode, which indicates the cohesive deformation between the Cr_2_AlC coating and TiBw/Ti6Al4V composite. The conformal cracking mode is the result of the excellent plastic deformation ability of MAX phases at room temperature. The formation of Cr_2_AlC coatings debris acts as a stress riser and causes an increase in the friction coefficient. After reaching the maximum load at the end of scratch, the coating buckling cracks and spallation was observed [82]. Therefore, the Cr_2_AlC coatings exhibit excellent mechanical integrity and elastic recovery.

## 4. Conclusions

In this study, Cr_2_AlC MAX phase coatings were successfully deposited on TiBw/Ti6Al4V composite through the HiPIMS and DCMS at different average power. The microstructure and mechanical properties of Cr_2_AlC coatings are comparatively studied in this work. The XRD results indicate that the as-deposited Cr-Al-C coatings were amorphous, which, upon annealing, crystallized into polycrystalline Cr_2_AlC MAX phase. The SEM results revealed that the morphology of Cr_2_AlC coatings produced by DCMS consists of typical columnar grains of nearly 250–500 nm. In contrast, the microstructure of coatings is improved in HiPIMS, and coatings possess fully dense, smooth, and column-free morphology. Increasing the pulse width and frequency is beneficial in increasing the average power in HiPIMS, thereby effectively increasing the deposition rate and the gas temperature around the substrate. The highest deposition rate and gas temperature of 56.3 nm/min and 195 °C were achieved at P_ave_ = 440 W. Moreover, the surface roughness of coatings decreases at a higher average power, and the surface roughness changed from 1.27 nm to 0.49 nm. The formation of defect-free coatings is an open challenge because the surface of coatings deposited by HiPIMS consists of surface defects (craters, pinhole, and pit). The Cr_2_AlC coatings with column-free morphology showed better hardness and modulus, and values of H/E and H^3^/E^2^ implied an excellent toughness and wear resistance compared with the DCMS and other PVD techniques, and the coatings exhibited maximum hardness and modulus of 19.7 GPa and 286 GPa, respectively. The fracture toughness and scratch test showed the good mechanical integrity of Cr_2_AlC coatings on TiBw/Ti6Al4V composite, and the critical load of 12.6 N was observed. Hence, the plasma density, temperature around the substrate, and average power can be controlled in HiPIMS easily, which is beneficial for achieving coatings with desirable properties. The dense and column-free microstructure of Cr_2_AlC coatings prepared by HiPIMS yielded the combined high hardness, good fracture toughness, and superior wear resistance, which will benefit the potential application of TiBw/Ti6Al4V composite in abrasive wear conditions. Moreover, it is believed that the denser morphology of Cr_2_AlC coating will exhibit enhanced oxidation and corrosion resistance, which will be investigated in our future work.

## Figures and Tables

**Table 1:** High-power impulse magnetron sputtering (HiPIMS) and dc magnetron sputtering (DCMS) deposition parameters for Cr_2_AlC coatings on TiBw/Ti64 composite.

Deposition Method	Voltage (V)	Frequency (kHz)	Pulse Width (μs)	Pressure (Pa)	P_ave_ (W)	P_peak_ (kW)
HiPIMS	600	1.2	40	0.5	240	6.6
	600	1.4	40		280	6.6
	600	1.6	40		320	6.6
	600	1.5	20	0.5	110	5.9
	600	1.5	40		300	6.6
	600	1.5	60		440	7.1
DCMS	330			0.5	112	

**Table 2:** Lattice parameters (a, c), crystal size (D), dislocation density (δ), and micro strains (ɛ) were calculated from the X-ray diffraction (XRD) data of the coatings prepared by DCMS and HiPIMS in comparison with the lattice parameter calculated by DFT-generalized gradient approximation (GGA) of bulk Cr_2_AlC MAX phase.

Process	P_ave_ (W)	a (Å)	c (Å)	c/a (Å)	V (A^3^)	D (nm)	δ × 10^−3^ (nm^−2^)	ɛ × 10^−3^ (%)	Ref.
DCMS	112	2.835	12.859	4.535	89.504	13.872	7.35 + 1.9	7.45 + 2.9	This work
HiPIMS	110	2.851	13.037	4.572	91.798	14.870	6.99 + 1.3	5.10 + 2.2	
	240	2.848	12.871	4.519	90.427	13.507	6.43 + 2.0	5.76 + 1.7	
	280	2.847	12.863	4.518	90.310	13.358	6.81 + 1.2	6.80 + 2.1	
	300	2.847	12.860	4.517	90.303	13.006	3.37 + 1.0	6.96 + 1.4	
	320	2.818	13.043	4.628	89.730	12.883	4.92 + 1.4	6.96 + 2.8	
	440	2.808	12.875	4.585	87.908	12.575	5.33 + 0.8	7.05 + 2.4	
DFT		2.847	12.793	4.493	89.804				This work
DCMS		2.85	12.93	4.536					Exp [20]
DFT		2.86	12.82	4.48	90.813				Theo [49]
HIP		2.85	12.81	4.49	90.109				Exp [50]

**Table 3:** Deposition parameters, defect size, and area distribution along with defect type on the coating surface prepared by HiPIMS.

Average Power (W)	Frequency (kHz)	Pulse Width (μs)	Defect Size (µm)	Defect Area (%)	Remarks
240	1.2		3.13 ± 1.51	4.94	pit, pinhole
280	1.4	40	1.33 ± 0.51	4.70	pinhole
320	1.6		0.66 ± 0.29	2.35	pinhole
110		20	None	None	None
300	1.5	40	3.73 ± 1.47	9.30	craters, pit
440		60	1.37 ± 0.30	5.17	craters

## Data Availability

The data presented in this study has not been published in total or in part elsewhere.
